# Supplementary material for: On the gene expression landscape of cancer
Source: PLoS One. 2023 Feb 21;18(2):e0277786. doi: 10.1371/journal.pone.0277786 (PMC9942972; doi:10.1371/journal.pone.0277786)
Supplement: S1 File — 1.1) Fraction of over- and under-expressed genes in the 15 tumors under study. 1.2) The contribution of genes to the unitary vector along PC1. 1.3) The proximity of tumors in GE space and the number of shared genes. 1.4) Stages in the evolution of tumors. 1.5) Pan-cancer genes and their characteristics. 1.6) Range of expression values in a typical TCGA data file. 1.7) Differentially expressed genes and top pathways. 1.8) Clustering analysis based on S2 Table. (PDF) [file pone.0277786.s001.pdf]

Supporting Information: On the gene expression landscape of cancer

Augusto Gonzalez[1,2], Dario A. Leon[3,2,*], Yasser Perera[4,5], Rolando Perez[1,6]

[1]University of Electronic Sciences and Technology of China, Chengdu, People Republic of China
[2]Institute of Cybernetics, Mathematics and Physics, Havana, Cuba
[3]Department of Mechanical Engineering and Technology Management, Norwegian University of Life Sciences, Ås, Norway
[4]China-Cuba Biotechnology Joint Innovation Center, Yongzhou, People Republic of China
[5]Center of Genetic Engineering and Biotechnology, Havana, Cuba
[6]Center of Molecular Immunology, Havana, Cuba
* dario.alejandro.leon.valido@nmbu.no

Keywords: Cancer Expression Profile, Cancer Attractor, Normal Tissue - Cancer Borderline

SI1 Fraction of over- and under-expressed genes in the 15 tumors under study.

For each tumor, we extract from the unitary vector along PC1 the 2500 genes with the largest amplitudes. In Fig. S1, the fraction of over- and under-expressed genes in these sets are drawn.

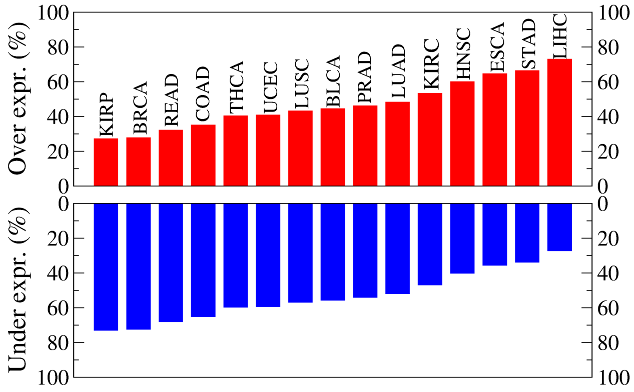

Figure S1: Fraction of over- and under expressed genes in the 15 tumors under study.

SI2 The contribution of genes to the unitary vector along PC1.

Taking PRAD as an example, the components of the unitary vector along PC1 are sorted with regard to their absolute values in order to draw Fig. S2. Notice that the first 2000 largest components exhibit a scaling, which can be taken as a manifestation of complex behavior in the gene regulatory network [1]. After that, the amplitudes suddenly decays to zero.

Let us stress that this number, 2000, is similar to the number of differentially expressed genes. In the main manuscript we have used the largest 2500 components of the vector in order to define a ranking of genes and compare tumors.

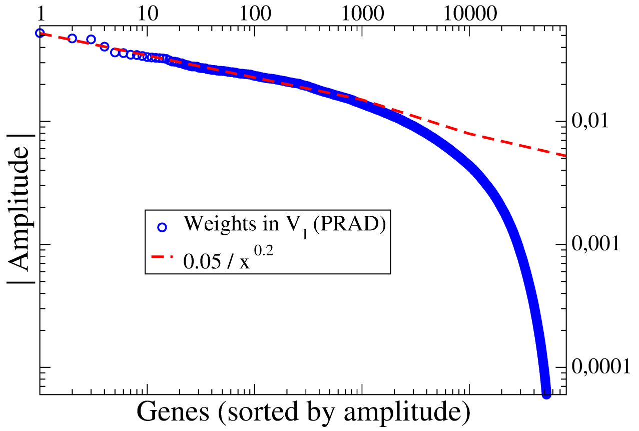

Figure S2: The components of the unitary vector along PC1 in PRAD, sorted according to their absolute values. Notice the scaling behavior in the largest 2000 components.

SI3 The proximity of tumors in GE space and the number of shared genes.

We show in Fig. S3 a correlation, indicating that the distance between two tumor centers is an actual indication of closeness. That is, the smaller the distance the larger the number of shared genes between the

two tumors.

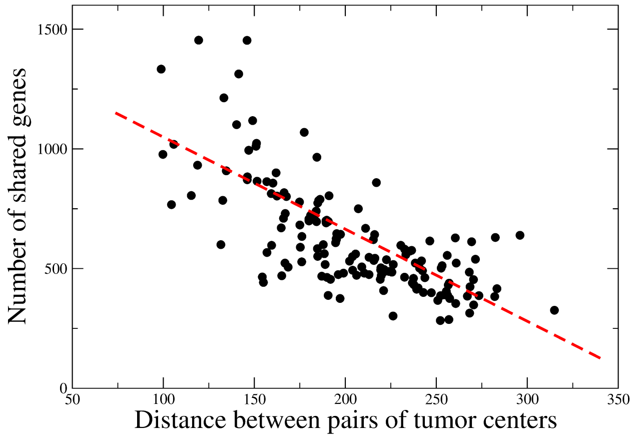

Figure S3: The inverse correlation between the distance between pairs of tumors and the number of shared differentially expressed genes. A fit with the inverse square root of the distance is added as a guide to the eye.

SI4 Stages in the evolution of tumors.

We have plotted in Fig. S4 the stages in the evolution of tumors in the cases of LIHC and LUSC.

SI5 Pan-cancer genes and their characteristics.

A supplementary Table S1 in *.xls* format is available online. 49 genes are differentially expressed in 11 tumors, whereas 6 common genes are found in the 15 studied cases. Notice that we are seeking for common genes in the first 2500 genes in the ranking for each tumor.

SI6 Range of expression values in a typical TCGA data file.

In Fig. S5, we plot expression values from a PRAD file. The x axis represents genes (sorted according to the expression values), whereas the y axis is the expression. Notice that the median for the expression is around 0.01. Instead of modeling the expression and defining a p-value, we use the simpler trick of adding a constant 0.1 to the expression, which leads to regularized differential expressions near one for all genes below the median.

SI7 Differentially expressed genes and top pathways.

A supplementary Table S2 in *.xls* format is available online containing information on differentially expressed genes and top pathways. We use the 28 top pathways of the Reactome database [2]. The number of calls to genes is indicated in the 2nd column. For each tumor, we check how many of these calls correspond to genes in the first 2500 genes in the ranking, and compute the fraction (%) of the total number of calls in the pathways. This is not an enrichment, but a frequency analysis. It provides an additional measure of closeness between tumors. That is, the more processes or pathways they share the closer they are.

SI8 Clustering analysis based on Suppl. Table S2.

Fig. 5 and Table 2 of the main text allow us to group tumors by the relative distance in the PC coordinates or by the number of common expressed genes respectively. A similar analysis can be performed with the 28 pathways presented in Suppl. Table S2, by searching for the common ones. For this purpose we have used

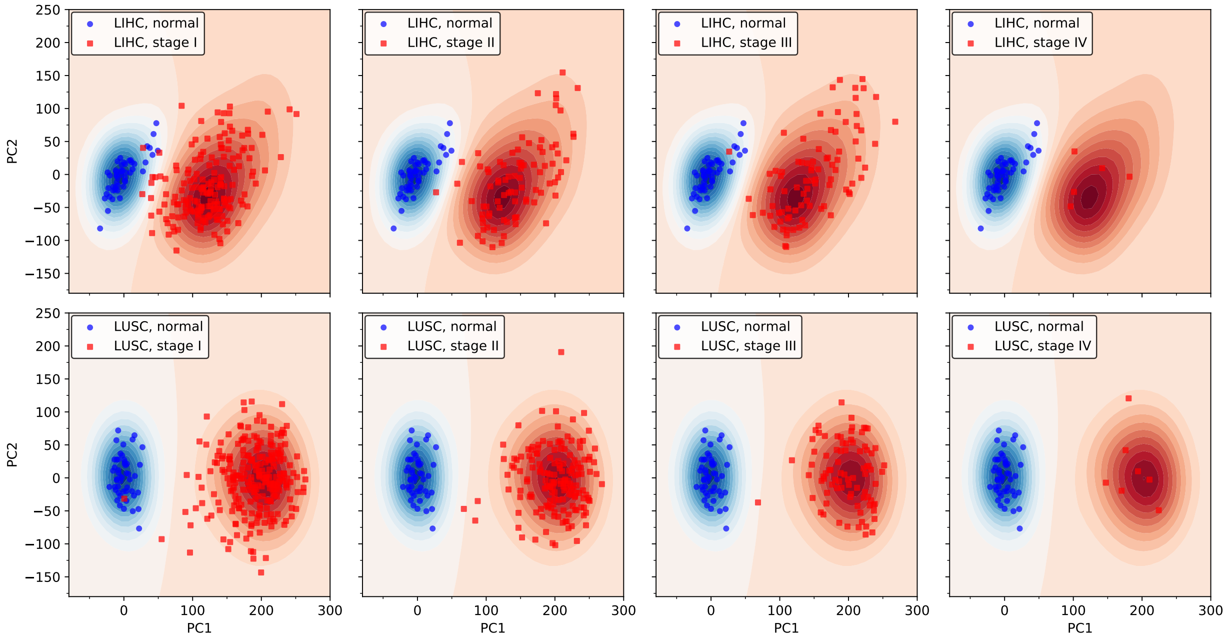

Figure S4: Stages in the evolution of tumors in LIHC and LUSC. The panels are analogous to the ones of KIRC in Fig. 4 of the main manuscript.

several linear and non-linear methods for data-dimensionality reduction including PCA, low-rank matrix factorization, isometric mapping, locally linear embedding and latent semantic analysis method. As an example, in Fig. S6 we plot the first 2 components corresponding to the later method. In general, the results of the main 2 components coming from the different methods cluster in similar groups of tumors. There are some similarities with the data in Fig. 5, from which LIHC, KIRC, KIRP, PRAD and THCA can be grouped. In the case of Fig. S6 the corresponding group is integrated by COAD, KIRC, KIRP, PRAD, READ and THCA. However, a further analysis is necessary in order to asses the relation between expressed genes and pathways.

References

[1] Didier Sornette. "Probability Distributions in Complex Systems". In: *Encyclopedia of Complexity and Systems Science*. Ed. by Robert A. Meyers. New York, NY: Springer New York, 2009, pp. 7009–7024. ISBN: 978-0-387-30440-3. DOI: https://doi.org/10.1007/978-0-387-30440-3_418.

[2] Bijay Jassal et al. "The reactome pathway knowledgebase". In: *Nucleic Acids Research* 48.D1 (2019), pp. D498–D503. DOI: https://doi.org/10.1093/nar/gkz1031.

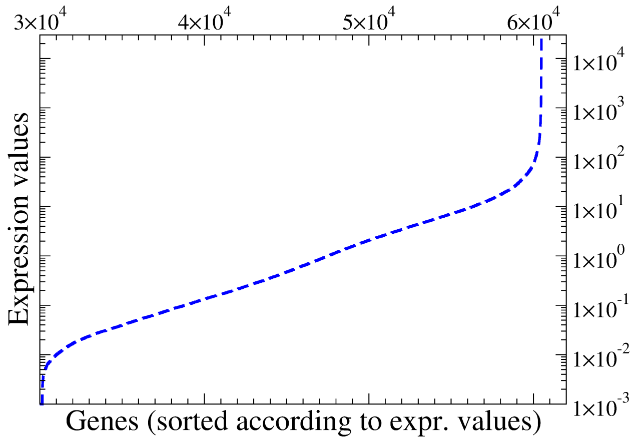

Figure S5: Range of values in a typical PRAD data file. Roughly half of the 60483 genes are not transcribed.

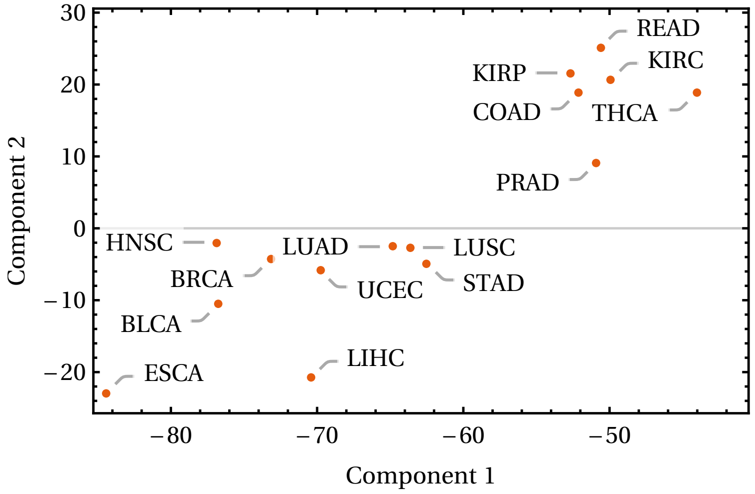

Figure S6: Dimension reduction of the data of Suppl. Table S2 by a latent semantic analysis method.
