# Supplementary material for: On the gene expression landscape of cancer
Source: PLoS One. 2023 Feb 21;18(2):e0277786. doi: 10.1371/journal.pone.0277786 (PMC9942972; doi:10.1371/journal.pone.0277786)
Supplement: Attachment — Submitted filename: plosone_response_vT.pdf [file pone.0277786.s004.pdf]

Dear Dr. Elingarami Sauli,

Please find enclosed a revised version of the paper, *On the gene expression landscape of cancer* by Augusto Gonzalez, Dario A. Leon, Yasser Perera and Rolando Perez.

We would like to express our gratitude for the interest in our manuscript, the careful reading and the comments and suggestions made by the referees. Below, we have included our detailed responses to all the comments. We have adopted the format of the journal, added three new references, [50],[52] and [53], and highlighted all the changes in the new version.

We hope that this revised version of the manuscript can be considered for publication in PLOS ONE.

Regarding the financial disclosure, we would like to update them by clarifying the additional requirements:

The funders had no role in study design, data collection and analysis, decision to publish, or preparation of the manuscript. Augusto Gonzalez received his salary from the Institute of Cybernetics, Mathematics and Physics, Havana, Cuba. Dario A. Leon received his salary from the Norwegian University of Life Sciences, Ås, Norway. Yasser Perera received his salary from the Center of Genetic Engineering and Biotechnology, Havana, Cuba. Rolando Perez received his salary from the Center of Molecular Immunology, Havana, Cuba. In addition, Dario A. Leon received financial support for publication from the Norwegian University of Life Sciences, Ås, Norway.

The authors have declared that no competing interests exist.

On behalf of all the authors

Dario A. Leon

Reviewer 1:

This manuscript is interesting and thought-provoking in that it addresses "grand" questions of cancer from a higher-order viewpoint, rather than ever narrower questions in ever more detail as most papers seem to do nowadays. However, the writing could be clearly improved and be more concise, precise and use a more scientific language. There are many "filling" words and sentences that can be omitted without losing information.

Authors Reply:

We thank the reviewer for his/her careful reading of the manuscript and his/her positive comments and suggestions.

We have rephrased some sentences along the hole manuscript and added more phrases to make the text clearer and more compressible. All the changes are highlighted in the new version.

Reviewer 1:

The following major and minor points should be addressed: Major points 1. The first two paragraphs of the Results section could be considerably shortened. Parts of it should be transferred to the Methods section, and/or covered by references. It is not necessary to explain in this paper how RNA-seq works, and it is not part of this manuscript‘s results. The authors state "In the Methods section, the used PCA methodology is briefly explained". This statement is sufficient for the Results section, further details on their PCA methodology should be in the Methods.

Authors Reply:

We thanks the referee for his/her suggestions. We have shortened the two paragraphs and moved not relevant information to the Method section.

Reviewer 1:

2. Figure 2 and the corresponding Text shows "a few thousand of genes" differentially expressed between tumors and normal tissue, and the magnitude of this differential expression is at least two-fold, and up to ¿100-fold. This is unusual, since similar previous analyses show fewer differentially expressed genes, and a lower magnitude of the differences.

Are all these differences statistically significant (by t-test or similar test statistics)? Have the authors properly normalized the gene expression data (per-gene and per-sample normalization)?

Authors Reply:

We thank the referee for this comment. In the new version of the manuscript we explain in more detail the normalization and the used methodology. Our simple procedure discards statistically irrelevant genes (its differential expression becomes 1 and the log fold variation, which enters the principal component analysis, becomes 0). Genes with high differential expression, highlighted by the methodology, have always a biological meaning.

Here, we would like to show data for PRAD as an example. The 1st figure is similar to Fig. 3 of our manuscript. There are only two genes (dlx1 and pca3) with differential expression higher than 20. There are also around 1000 genes with $e_{diff} > 2$, and around 5000 genes with $e_{diff} > 1.5$. Note that a study of statistically significant differentially expressed genes in PRAD identifies around 11,000 genes (doi: 10.1371/journal.pone.0145322).

In paper doi: 10.1038/s41598-021-87764-0, we relate this relative high GE rearrangement (that is, thousands of diff expressed genes) to a discontinuous transition between the normal and the tumor state.

The 2nd figure plots sample data for pca3 (a known PRAD marker) in age groups. Mean geometric averages are represented in red. The NY group is taken as reference for normalization. This 2nd fig contributes with 3 point to the 1st figure: that is, one for each of the NO, TY and TO curves.

Reviewer 1:

3. Legends to Figures and Tables should explain what is shown in corresponding Figures and Tables in more detail and with more precision, e.g. all abbreviations should be defined (or at least refer to an item where those are defined), and x- and y-axes should be well defined (e.g. x-axis in Figure 3 is labelled "Differential Expression" – are these fold-changes? Probably yes, but this should be defined!).

Authors Reply:

We thanks the referee for his/her comments. We have added more details to the caption

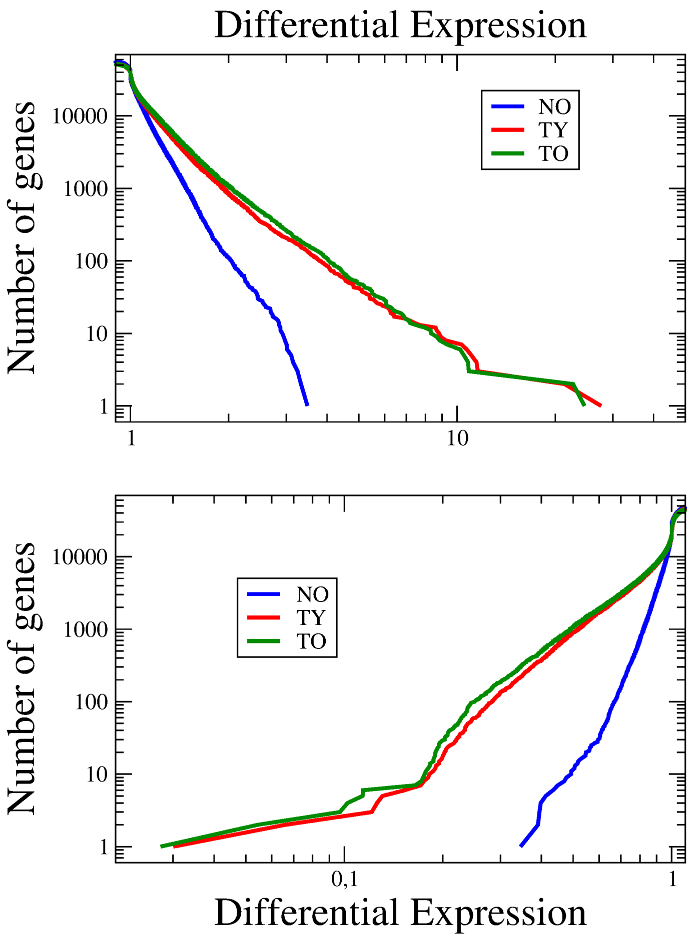

FIG. 1: PRAD

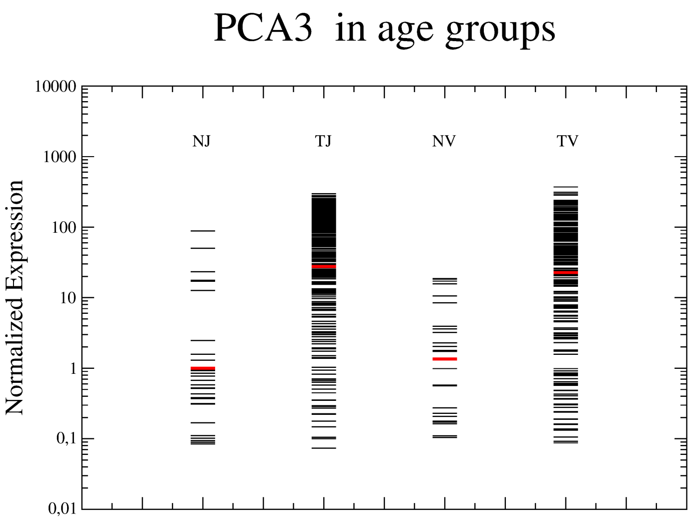

FIG. 2: PRAD

of the figures and tables. In particular, the x axis in Fig. 3 is properly the differential expression. The log-log plot is simply to show the Paretto law in the tail distribution function.

Reviewer 1:

4. In statement 4, second part (page 6), the effect of tumor stage (I, II, II, IV) on gene expression of tumors is discussed, and it is assumed that higher-stage tumors should be progressed further on the normal-tumor gene expression axis. However, whereas expression is analysed in the primary tumor, the tumor stage is not a property of the primary tumor alone, e.g. stage IV commonly means that distant metastasis are present, and lymph node metastasis also play a key role in determining the tumor stage (which may or may not affect the gene expression state of the primary tumor analysed here). Probably the tumor size or tumor grade (both a property of the primary tumor) would be a better parameter than tumor stage to address this question. In any case, it should be defined (or a reference provided), how tumor stage I-IV are defined in KIRC, and this potential limitation should be discussed.

Authors Reply:

We recognize the limitations to draw "attractor basins" even in the context of advanced tumors stages (III-IV) by only surveying the primary tumor. However, our assumption here is that in primary tumors some information remains in their gene expression profiles, concerning the original seeding of the distant metastasis, and/or that the process of cell invasion and dissemination from this primary site is continuous.

In the same line, other groups have also extracted and proposed prognostic signatures for advanced KIRC stages by studying primary tumor samples. See, for example:

https://doi.org/10.3389/fbioe.2019.00270

https://doi.org/10.1111/cbdd.14141

https://doi.org/10.3389/fonc.2022.912155

Reviewer 1:

Minor points 1. "Statement 1-5" in Results: Are these results, conclusions or hypothesis? More precision in wording is needed.

Authors Reply:

We have clarified at the beginning of the Results section, that the statements refer to results/conclusions, following straightforwardly from the analysis of the GE data.

Reviewer 1:

2. Statement 1 (page 4): the authors refer to "the left panel" and "the right panel" of Figure 1. They probably mean the upper panel and the lower panel.

Authors Reply:

We thanks the referee for pointing this out, we have corrected it.

Reviewer 1:

3. This reviewer recommends to omit the scale lines within the panels in Figure 2, they are distracting and may cause problems during printing and photo-copying.

Authors Reply:

Thanks the reviever for this comment, we have removed the grids.

Reviewer 1:

4. Page 4 bottom: "the rest of the 60000 genes". The authors should explain how they arrive at this number. There are about 25000 human protein-coding human genes, were additional genes (miRNAs, long nc RNAs etc.) included in this number? The authors should provide their (or the TCGA's) definition of "gene".

Authors Reply:

The referee is right, TCGA data involves measurement of the expression for 60487 RNA- and protein-coding genes in the Ensembl notation. We have made explicit this point in the text.

Reviewer 1:

5. Page 6: "Well defined regions in expression space is a fact in favor of the attractor paradigm of cancer" could be replaced by "Well defined regions in expression space support

the attractor paradigm of cancer".

Authors Reply:

We thanks the referee for pointing this out, we have corrected it accordingly.

Reviewer 1:

6. "sub-expression" should rather be termed "under-expression", as opposed to over-expression, or "up-regulation" and "down-regulation" should be used (throughout the manuscript).

Authors Reply:

We thanks the referee for pointing this out, we have uniformized the notation.

Reviewer 1:

7. The Formatting of the References apparently does not adhere to PLOS One style, in particular, the full first names of authors are provided.

Authors Reply:

We thanks the referee for pointing this out, we have corrected the format of the references and the manuscript accordingly.

Reviewer 2:

PCA has been used to analyze GE data from TCGA dataset. In detail, the results of the PCA of 15 different tumors seem to support 5 different facts or statements. Overall, the manuscript is well written and PCA well performed.

Authors Reply:

We thanks the referee for his/her very positive comments.

Reviewer 2:

I have some minor comments: - As authors stated: "results are simple, general and unbiased, in the sense that no modeling or elaborated mathematical treatments are used".

It would be better to describe the possible limits of this approach. - For example, could we use the pan-cancer genes (i.e, the genes common to all 15 tumors) in therapies designed to act in this group of tumors? Are these druggable targets?

Authors Reply:

We thanks the referee for raising these points. We have stressed in the new version of the manuscript that the results of our PCA analysis are limited mainly by the number of available samples. The second raised question on the pan-cancer genes is really interesting, but beyond the scope of the paper.

Druggable targets are defined as those molecules (i.e. proteins, peptides, nucleic acids) which levels and/or specific activities can by modulated by a drug, which can consist of a small molecular weight chemical compound (SMOL) or a biologic (BIOL), such as an antibody or a recombinant protein. Two issues need to be considered on the putative druggability of these reported pan-cancer genes: 1) Gene expression changes highlighted here need to be corroborated at the protein level if we attempt to develop a target therapy against the final protein product. 2) Usually, protein down-regulation and/or diminished activity, are harder to tackle than protein over-expression and/or activity inhibition. However, as described below, there are experimental and approved drugs for 2/3 up-regulated pan-cancer genes, whereas there are recent reported drugs aiming at the three down-regulated genes:

a) MMP11 (Up, Matrix Metallopeptidase 11): at least two experimental drugs are reported at https://www.genecards.org/cgi-bin/carddisp.pl?gene=MMP11

b) C7 (Down, Complement Component C7): at least one experimental drug is reported at https://www.genecards.org/cgi-bin/carddisp.pl?gene=C7&keywords=C7

c) ANGPTL1 (Down,Angiopoietin Like 1): one inferred drug is reported at https://www.genecards.org/cgi-bin/carddisp.pl?gene=ANGPTL1&keywords=ANGPTL1#drugs_compounds

d) UBE2C (Up, Ubiquitin Conjugating Enzyme E2 C): at least three approved drugs are reported at https://www.genecards.org/cgi-bin/carddisp.pl?gene=UBE2C&keywords=UBE2C#drugs_compounds

e) IQGAP3 (Up, IQ Motif Containing GTPase Activating Protein 3): no reported or inferred drug so far at https://www.genecards.org/cgi-bin/carddisp.pl?gene=IQGAP3&keywords=IQGAP3

f) ADH1B (Down, Alcohol Dehydrogenase 1B (Class I), Beta Polypeptide): there are more than 5 approved drugs and another handful under developmental stages.

Of note, in a former comprehensive redefinition of the druggable genome (doi: 10.1126/scitranslmed.aag1166), the authors stratified the druggable gene set into three tiers corresponding to position in the drug development pipeline. Tier 1 (1427 genes) included efficacy targets of approved small molecules and bio-therapeutic drugs as well as clinical-phase drug candidates. Tier 2 was composed of 682 genes encoding targets with known bioactive drug-like small-molecule binding partners as well as those with $\geq 50\%$ identity (over $\geq 75\%$ of the sequence) with approved drug targets. Tier 3 contained 2370 genes encoding secreted or extracellular proteins, proteins with more distant similarity to approved drug targets, and members of key druggable gene families not already included in tier 1 or 2. Furthermore, the more frequent Pfam-A domain content in three tiers of druggable genes were also described.

After inspecting this data we noted that ADH1B was positioned into Tier 1, whereas MMP11, C7 and ANGPTL1 were located to Tier 3B. Overall, only IQGAP3 remains elusive as a putative druggable target within the six pan-cancer genes. IQGAP3 contains the Rho_GTPase_activation_prot domains not included in tiers 1-3.
